# Anthropometric and Biochemical Characteristics of Polycystic Ovarian Syndrome in South Indian Women Using AES-2006 Criteria

**DOI:** 10.5812/ijem.12470

**Published:** 2014-01-05

**Authors:** Sujatha Thathapudi, Vijayalakshmi Kodati, Jayashankar Erukkambattu, Anuradha Katragadda, Uma Addepally, Qurratulain Hasan

**Affiliations:** 1 Department of Genetics and Molecular Medicine, Vasavi Medical and Research Centre, Khairatabad, Hyderabad, India; 2 Department of Genetics and Molecular Medicine, Geneticist and Research Coordinator, Vasavi Medical and Research Centre, Khairatabad, India; 3 Department of Pathology, Kamineni Academy of Medical Sciences and Research Centre, LBnagar, Hyderabad, India; 4 Department of Gynaecology, Anu’s Fertility Centre, Somajiguda, Hyderabad, India; 5 Department of Biotechnology, Research Coordinator, Jawaharlal Nehru Technological University, Kukatpally, Hyderabad, India; 6 Senior Scientist and Geneticist, Kamineni Academy of Medical Sciences and Research Centre and Vasavi Medical and Research Centre, Hyderabad, India

**Keywords:** Polycystic Ovarian Syndrome, Body Mass Index, HOMA Score, Insulin Resistance

## Abstract

**Background:**

Polycystic ovarian syndrome (PCOS) is one of the most common endocrine conditions affecting women of reproductive age with a prevalence of approximately 5-10% worldwide. PCOS can be viewed as a heterogeneous androgen excess disorder with varying degrees of reproductive and metabolic abnormalities, whose diagnosis is based on anthropometric, biochemical and radiological abnormalities. To our knowledge, this is the first study investigating the anthropometric, biochemical and ultrasonographic characteristics of PCOS in Asian Indians of South India, using the Androgen Excess Society (AES-2006) diagnostic criteria.

**Objectives:**

To assess anthropometric, biochemical and ultrasonographic features of PCOS subgroups and controls among South Indian women using the AES-2006 criteria.

**Materials and Methods:**

Two hundred and four women clinically diagnosed with PCOS, and 204 healthy women controls aged 17 to 35 years were evaluated. PCOS was diagnosed by clinical hyperandrogenism (HA), irregular menstruation (IM), and polycystic ovary (PCO). PCOS was further categorized into phenotypic subgroups including the IM+HA+PCO (n = 181, 89%), HA+PCO (n = 23, 11%), IM+HA (n = 0), and also into obese PCOS (n = 142, 70%) and lean PCOS (n = 62, 30%) using body mass index (BMI). Anthropometric measurements and biochemical characteristics were compared among the PCOS subgroups.

**Results:**

The PCOS subgroups with regular menstrual cycles (HA+PCO), had more luteinizing hormone (LH), follicle stimulating hormone (FSH), fasting glucose, fasting insulin, and high insulin resistance (IR) expressed as the Homeostasis Model Assessment (HOMA) score, compared with the IM+HA+PCO subgroups and controls. Similarly, the obese PCOS had high BMI, waist to hip ratio (WHR), fasting glucose, LH, LH/FSH, fasting insulin, HOMA score (IR), and dyslipidemia, compared with lean PCOS and controls. Unilateral polycystic ovary was seen in 32 (15.7%) patients, and bilateral involvement in 172 (84.3%) patients. All the controls showed normal ovaries.

**Conclusions:**

Anthropometric, biochemical, and ultrasonographic findings showed significant differences among PCOS subgroups. The PCOS subgroups with regular menstrual cycles (HA+PCO), had high insulin resistance (IR) and gonadotropic hormonal abnormalities, compared with the IM+HA+PCO subgroups and controls.

## 1. Background

Polycystic ovarian syndrome (PCOS) is one of the most common endocrine dysfunctions in women of reproductive age with a prevalence of approximately 5-10% worldwide (1-5), characterized by irregular menstruation (IM), obesity, hyperandrogenism (HA) and polycystic ovary (PCO). After the first description of PCOS by Stein and Leventhal, the diagnostic criteria of PCOS have evolved over the years. The 1990 National Institutes of Health (NIH) conference proposed the diagnostic criteria of oligo/anovulation and biochemical and/or clinical hyperandrogenism. In 2003, Rotterdam conference, organized by the European Society of Human Reproduction and Endocrinology (ESHRE) and American Society of Reproductive Medicine (ASRM) broadened the definition of PCOS by adding PCO morphology, which divided PCOS into four subtypes: IM/PCO/HA, IM/PCO, IM/HA, and HA/PCO. The Rotterdam criteria do not delineate the essential features of PCOS. In 2006, the Androgen Excess Society taskforce on the phenotypes of PCOS emphasized HA as the cornerstone of PCOS (1, 2) and excluded the IM/PCO subgroup. The second criterion essential to diagnose PCOS according to the AES is either anovulation or polycystic ovarian morphology. The principle features of PCOS are insulin resistance and hyperandrogenism (5). The role of obesity as a contributing factor in the development of PCOS is widely accepted (6), and particularly the abdominal phenotype (central obesity) may be responsible for IR and associated hyperinsulinemia in women with PCOS (7, 8). Insulin resistance can augment hyperandrogenism. The pathophysiology of PCOS is still not clear, and medical care of these patients has been limited to symptomatic control and infertility. To our knowledge, no study has assessed the different clinical, biochemical and metabolic characteristics of PCOS subtypes and healthy controls among Asian women, using the AES-2006 criteria, until now.

## 2. Objectives

The aim of the present study was to determine different clinical, biochemical and metabolic characteristics in PCOS subgroups and healthy controls among South Indian women using the AES-2006 criteria.

## 3. Patients and Methods

This study was approved by the Institutional Ethics Committee, and informed written consent was obtained from all subjects. This prospective case-control study included 204 consecutive PCOS patients from different Obstetrics and Gynecology centers, and the general population from July 2011 to January 2013. Patients were diagnosed using the AES-2006 criteria: 1. Hyperandrogenism, clinical or biochemical and either 2. Oligo/anovulation or 3. PCOM. All the subjects underwent transvaginal ultrasound or transabdominal ultrasound examination in the follicular phase to evaluate ovarian morphology, and any other lesions in the pelvic region. PCOS patients were subgrouped into HA+IM+PCO, HA+PCO and HA+IM based on the AES criteria, and also into obese and lean PCOS based on BMI and the Asia pacific definition of obesity (≥ 25 and < 25 kg/m^2^, respectively).

Exclusion criteria: women with congenital adrenal hyperplasia, androgen-secreting neoplasms, androgenic/anabolic drug use or abuse, Cushing’s syndrome, syndromes of severe insulin resistance, thyroid dysfunction, and hyperprolactinemia were excluded from the study.

Two hundred and four controls were studied during the same period. They visited the health-care center in a super specialty hospital for comprehensive medical checkups, and did not show hirsutism, acne, or male-type baldness. All of them had regular menstrual cycles ranging 27 to 35 days, and none of them fulfilled any of the AES-2006 criteria. All control subjects underwent an ultrasonographic examination by a gynecologist, and women who had any pathologic findings or polycystic ovaries were excluded from the study. Women, who consumed medication that might affect endocrinological or metabolic changes, were also excluded from the control group.

Definitions: waist circumference (WC); middle circumference between the iliac crest and the lateral coastal margin, hip circumference (HC); largest measurements over the buttocks, waist-to-hip ratio (WHR); waist circumference divided by hip circumference, oligomenorrhea; absence of menstruation for >35 days, amenorrhea; no menstruation for more than 6 months, clinical hyperandrogenism; modified Ferriman-Gallwey (mFG) score > 8 with or without acne and/or androgen alopecia (9). Hirsutism was scored by studying terminal hair in nine body areas (upper lip, chin, chest, upper and lower abdomen, upper arms, thighs, and upper and lower back). The occurrence of acne was recorded by areas of distribution and degree of affection with lesions (papules, cysts, scars, or abscesses) categorized simply as mild, moderate and severe. Acanthosis nigricans (AN); dark, velvety, skin thickening on the neck, axilla, and other sites such as face, chest and knuckles were recorded. 

AES/2006 definition criteria for PCOS requires the presence of hyperandrogenism (clinical and/or biochemical) excluding other causes of hyperandrogenism and either ovulatory dysfunction or polycystic ovarian morphology. 

The definition of polycystic ovarian morphology by ultrasound examination is the presence of >12 follicles with 2 to 9 mm diameter in the ovary. An ovarian volume >10 mL is also suggestive. Only one ovary consistent with PCO morphology is sufficient for the diagnosis (10). 

According to the WHO classification of obesity, the body mass index (BMI) is categorized into < 24.99 as normal, 25-29.9 as overweight, and >30 as obese. However based on the Asia-Pacific definition (7, 8, 11-13) individuals can be subdivided into obese and lean, based on BMI > 25 and < 25, respectively. 

The International Diabetes Federation (IDF) definition of Metabolic syndrome (MetS) includes the presence of central obesity, WC > 80 cm, with any of the two following: hypertension > 130/85 mmHg, FPG >5.6 mmol/L, TG > 1.7 mmol/L, and HDL < 1.3 mmol/L (14). MetS, defined by the National Cholesterol Education Program’s Adult Treatment Panel III [ NCEP (ATP III)] report, (14) is presence of Three or more of the following risk factors: waist circumference (WC) > 88 cm, hypertension > 130/85 mmHg, fasting plasma glucose (FPG) > 6.1 mmol/L, triglyceride (TG) > 1.7 mmol/L, and high-density lipoprotein (HDL) < 1.3 mmol/L. 

Clinical findings: clinical history included a questionnaire-based interview regarding socio-demographic factors, detailed menstrual and obstetric history, onset and degree of clinical symptoms of PCOS, drug history, changes after treatment, dietary habits, and family history (family pedigree) of PCOD, diabetes, hypertension, and cardiovascular risk factors. Physical examination of BMI, WC, WHR, blood pressure, acne, hirsutism, alopecia, male pattern hair loss, acanthosis nigricans, transvaginal ultrasound examination in married women, and transabdominal ultrasound examination in unmarried women, were performed by gynecologists/ radiologists.

Biochemical and Hormonal findings: fasting plasma glucose (enzymatic colorimetric method), fasting insulin (ELISA method using DRG kits, The USA), thyroid stimulating hormone, LH, FSH (ELISA method using Omega diagnostics, The UK), serum cholesterol, triglycerides, HDL were measured using the enzymatic colorimetric assay (Coral & Merck kits, Micro lab 300, Merck). Laboratory controls were used to check the accuracy and precision of the analyzer, reagents and assay results.

Data and statistical analysis: BMI = weight/height^2^ (kg/m^2^). The Homeostatic model assessment for insulin resistance (HOMA-IR) was calculated by using the formula: fasting serum insulin (µU/mL) × Fasting plasma glucose (mg/dL) / 405 (15). Statistical analysis (one-way ANOVA) of all the data was performed using the Graphpad Instat online software. A P value of < 0.05 was considered significant. 

## 4. Results

Phenotypic (subgroup) analysis using AES criteria: for the present study, based on the AES criteria, we had only two subgroups, the IM+HA+PCO group (n = 181, 89%), and the HA+PCO subgroup (n = 23, 11%), and no IM+HA subgroup.

The age range was 17 to 35 years for both patients and controls. The mean age of attaining menarche in PCOS patients was 13.03 + 1.25 years, and for controls was 14 + 1.5 years. The mean age of women in the control group (28 + 5.25) was similar to the mean age of PCOS subgroups. The mean BMI, WC, HC, W/H of PCOS subgroups were significantly higher (P < 0.0001) than the controls. Among PCOS subgroups, IM+HA+PCO showed more BMI, WC, HC, but the W/H was similar in the PCOS subgroups.

Central obesity: WC > 75 cm and W/H ratio > 0.85 cm indicates central obesity (12). The IM+HA+PCO subgroup showed 95% (n = 172) and HA+PCO 100% central obesity.

**Figure 1.**
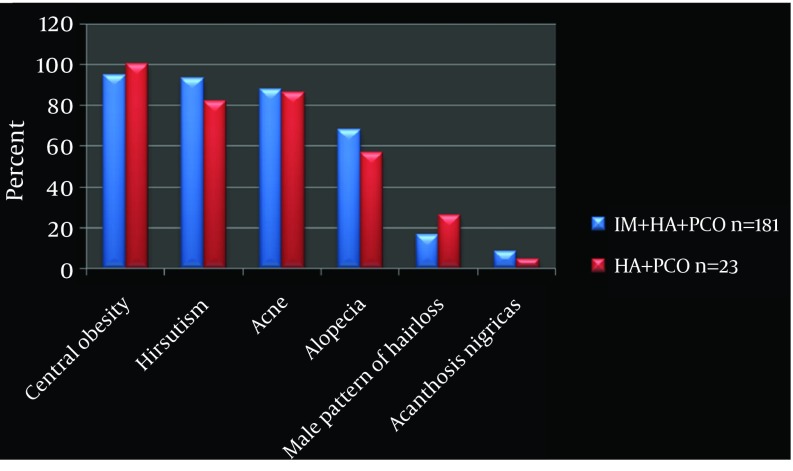
Clinical Features of Hyperandrogenism IM, Irregular Menstruation; HA, Hyperandrogenism; PCO, Polycystic Ovary; n, number

Irregular Menstruation (IM): In the IM+HA+PCO subgroup (n = 181, 89%), irregular menstruation was observed in all. The HA+PCO subgroup (n = 23, 1 1%) had regular menstrual cycles (Figure 1). 

Metabolic syndrome in PCOS subgroups: based on the definitions of MetS (NCEP, ATP-III, and IDF), 26% [N = 53, IM+HA+PCO (n = 50), and HA+PCO (n = 3)] fulfilled the criteria (Table 1). 

Personal history: in the IM+HA+PCO subgroup, 17 cases of HTN, 20 cases of DM and 2 cases of CAD were noted. In the HA+PCO subgroup, 3 cases of HTN and 2 cases of DM were noted.

**Table 1.** Comparison of Anthropometric and Biochemical Characteristics of Obese PCOS, Lean PCOS and Controls

Sample, No.	Parameter	Obese PCOS	Lean PCOS	Controls	P Values ^a^
**1^b^**	BMI ^c^, kg/mL	29.30 + 4.22	22.00 + 1.70	23.40+ 3.20	< 0.0001
**2^b^**	W/H	0.94 + 0.04	0.90 + 0.03	0.79 + 0.05	< 0.0001
**3^b^**	F Glucose, mg/dL	89.00 + 12.67	85.80+9.48	86.85 + 7.10	0.0470
**4 ^b^**	F Insulin, µU/mL	14.20 + 13.50	13.40 + 12.00	6.60 + 3.19	< 0.0001
**5^b^**	HOMA score	3.10 + 2.93	2.79 + 2.33	1.44 + 0.75	< 0.0001
**6**	TSH, mIU/mL	2.20 + 1.20	2.60 + 1.48	3.20 + 7.60	0.2338
**7^b^**	LH, mIU/mL	12.00 + 5.76	11.36 + 6.31	7.90 + 5.46	< 0.0001
**8^b^**	FSH, mIU/mL	5.26 + 1.80	5.80 +2.19	6.47 + 3.16	0.0001
**9^b^**	LH/FSH	2.40 + 1.00	2.10 + 1.44	1.50 + 1.20	< 0.0001
**10**	Chol, mg/dL	165.00 +31.00	160.00 + 29.00	162.70 + 33.00	0.5243
**11^b^**	HDL, mg/dL	41.00+ 9.80	39.00 + 8.50	45 + 13.40	0.0002
**12^b^**	TG, mg/dL	118.00+39.28	117 + 32	97 + 49	< 0.0001

^a^ ANOVA one way analysis –posttest if P value < 0.05

^b^ P values are significant

^c^ Abbreviations: BMI, Body mass index; W/H, waist/hip Fasting glucose and fasting insulin, TSH, Thyroid stimulating hormone; LH, Luteinizing hormone; FSH, Follicular stimulating hormone; HOMA score, Homeostatic model assessment score; Chol, cholesterol; HDL, high density lipoprotein; TG, Triglycerides; LDL, Low density lipoprotein; VLDL, very low density lipoprotein

Hyperandrogenism (HA): the IM+HA+PCO subgroup showed more hirsutism (93%) compared to the HA+PCO subgroup, (82%). Acne, alopecia, male pattern of hair loss and acanthosis nigricans were seen more in the IM+HA+PCO subgroup (88%, 68%, 16.5%, and 8.25%), compared to the HA+PCO subgroup (86%, 56.5%, 26%, and 4.3%) (Figure 1).

Polycystic ovary (PCO): all the PCOS patients had undergone baseline ultrasound scanning (transvaginal-196, transabdominal-8). In the IM+HA+PCO subgroup, 84.5% (n = 153) showed bilateral PCO, and 15.5% (n = 28) unilateral PCO. In the HA +PCO subgroup, 82% (n = 19) showed bilateral PCO and 18% (n = 4) unilateral PCO.

Infertility: of the 204 patients, eight were unmarried. In the remaining 196 patients, the IM+HA+PCO subgroup showed, 80% primary infertility, and 15.5% secondary infertility. The HA+PCO subgroup showed 82% primary infertility, and 18% secondary infertility. 

Biochemical Parameters: the hormonal and metabolic features of PCOS subgroups were compared with the controls (Table 2). The HA+PCO subgroup showed more LH, FSH, fasting glucose, fasting insulin, HOMA score, and HDL compared to the IM+HA+PCO subgroup. Significant P value of LH, LH/FSH, fasting insulin, HOMA score, HDL, TG, and VLDL was noted, between the PCOS subgroups and controls.

**Table 2.** Comparison of Mean Values of Anthropometric and Biochemical Characteristics in PCOS Subjects Using the AES Criteria

Sample, No.	Parameter	HA+PCO+IMC (n=181) ^a^	HA+PCO (n=23)a	Controls (n = 204) ^a^	P value ^b^ (PCOS Subgroups vs. Control)
**1**	Age, y	28.27 + 3.70	27.91 + 1.00	28.00 + 5.25	0.8631
**2 ^c^**	BMI ^d^, kg/m2	27.25 + 5.02	26.00 + 4.10	23.40 + 3.20	< 0.0001
**3 ^c^**	WC, cm	37.10 + 4.28	35.30 + 4.18	30.36 + 3.30	< 0.0001
**4 ^c^**	HC, cm	39.60 + 4.04	37.40 + 4.50	38.11 + 3.70	< 0.0001
**5 ^c^**	WHR	0.93 + 0.04	0.94 + 0.036	0.79 + 0.50	< 0.0001
**6**	TSH, mIU/mL	2.33 + 1.29	2.50 + 1.05	3.20 + 7.60	0.1664
**7 ^c^**	LH, mIU/mL	11.83 + 6.18	13.09 + 5.20	7.90 + 5.46	< 0.0001
**8**	FSH, mIU/mL	5.30 + 1.98	6.29 + 2.25	6.47 + 3.16	0.4864
**9 ^c^**	LH/FSH	2.41 + 1.35	2.16 + 0.67	1.50 + 1.20	< 0.0001
**10**	Fasting glucose, mg/dL	87.83 + 10.84	91.80 + 14.86	86.85 + 7.10	0.1574
**11 ^c^**	Fasting insulin, µU/mL	16.40 + 17.10	21.10 + 17.90	6.66 + 3.19	< 0.0001
**12 ^c^**	HOMA Score	3.80 + 3.59	4.80 + 4.00	1.44 + 0.75	< 0.0001
**13**	Chol, mg/dL	162.00 + 29.82	151.50 + 31.00	162.70 + 33.00	0.2620
**14 ^c^**	HDL, mg/dL	40.00 + 10.37	41.37 + 8.90	45.00 + 13.40	0.0002
**15 ^c^**	TG, mg/dL	128.82 + 45.00	105.00 + 35.60	97 + 49	< 0.0001
**16**	LDL, mg/dL	97.60 + 29.83	88.70 + 30.50	96.99 + 32.24	0.4233
**17 ^c^**	VLDL, mg/dL	25.45 + 8.79	21 + 7	20.82 + 11.79	0.0403

^a^ Data are shown as mean ± SD

^b^ P values were evaluated by one–way ANOVA with post test

^c^ Significant values (P is < 0.05)

^d^ Abbreviations: HA, hyperandrogenism; PCO, polycystic ovary; IM, Irregular menstruation

Family history of metabolic diseases: there were 75 patients with more than one affected family member. In the IM+HA+PCO subgroup, family history of DM (n = 42, 23%), HTN (n = 14, 8%), CAD (n = 7, 4%), DM+HTN+CAD (n = 42, 23%), DM+HTN (n = 36, 20%), DM+CAD (n = 4, 2%) and HTN+CAD (n = 2, 1%) were noted. In the HA+PCO subgroup, family history of DM (n = 8, 35%), HTN (n = 1, 4%), DM+HTN+CAD (n = 4, 17%) and DM+HTN (n = 5, 22%) were noted.

Family history of PCOD, late conception, infertility, and irregular menstrual cycles: in the IM+HA+PCO subgroup, 20 cases of PCOD, 71 cases of late conceiving, 20 cases of infertility, and 41 cases of irregular menstrual cycles were noted. In the HA+PCO subgroup, 3 cases each of PCOD, late conceiving and 2 each of infertility and irregular menstrual cycles were noted.

Anthropometric, biochemical differences betweenobese and lean PCOS: for this study, we had 141 (69%) cases of obese PCOS and 63 (31%) of lean PCOS. When the means were compared among these subgroups and controls, obese PCOS showed higher BMI, W/H, fasting insulin, HOMA score, LH, LH/FSH, HDL and TG, which were significant (P < 0.05) (Table 3). 

**Table 3.** Metabolic Syndrome Prevalence in PCOS Subgroups and Controls (NCEP ATP III, and IDF Criteria)

Parameters	IM+HA+PCO (n = 181)	HA+PCO (n = 23)	Controls (n = 204)
**WC ** ^**a**^ **≥ 88 cm/ ≥ 80 cm**	94/145	08/17	08/45
**TG ≥ 1.7 mmol/L (150 mg/dL)**	50	03	20
**HDL ≤ 1.3 mmol/L (50 mg/dL)**	163	21	160
**BP ≥ 130/85 mmHg**	18	04	-
**FB glucose > 6.1 mmol/L (101 mg/dL)**	15	02	-
**No of subjects fulfilling the criteria**	50 (28%)	03 (13%)	08 (4%)/20 (10%)

^a^ Abbreviations: MetS, Metabolic Syndrome; PCOS, Polycystic Ovarian Syndrome; NCEP ATP III, National Cholesterol Education Program Adult Treatment Plan III; IDF, International Diabetes Federation; IM, Irregular Menstruation; HA, Hyperandrogenism; PCO, Polycystic Ovary; WC, Waist Circumference; TG, Triglycerides; HDL, High Density Lipoprotein; FBG, Fasting Blood Glucose.

## 4. Discussion

Several reports on PCOS patients published earlier were related mainly to anthropometric, biochemical, and radiologic details. In the AES-2006 criteria (1, 2, 5), HA is the key feature of PCOS along with irregular menstruation and polycystic ovaries, and hence we reclassified the subgroups according to HA. Our data showed that the IM+HA+PCO showed different clinical and biochemical characteristics compared to HA+PCO and controls.. To our knowledge, this is the first study investigating PCOS patients in Southern India, using the AES-2006 criteria, which included family history with pedigrees and metabolic disorders. 

In our cohort of PCOS, 89% had irregular menstruation, and 11% had regular menstruation, which is different from the findings of Azziz et al. (1), meta-analysis of PCOS patients, which showed approximately 75% irregular menstruation, and 20% regular menstruation. The clinical features of hyperandrogenism were more evident in the IM+HA+PCO subgroup than in the HA+PCO group. The prevalence of hirsutism and acne were respectively 93% and 82% in the IM+HA+PCO subgroup, and 88% and 86% in the HA+PCO, and was more than a meta-analysis, which showed 60% hirsutism, and 15-20% acne (1, 2). The higher prevalence of hirsutism in our population is similar to the observation made by Wijeyratne et al. (15) among Southern Asians. In our study population, 96% of the patients showed polycystic ovaries, by transvaginal ultrasonography compared to 75% seen in the meta-analysis mentioned. The higher frequency of clinical features of irregular menstruation, hyperandrogenism, and polycystic ovaries can be explained by the strict inclusion criteria of our study population, and selection of the PCOS patients attending only the infertility clinics. 

Studies have shown that BMI differs across ethnic groups (7, 8, 12, 13). The role of obesity in the development of PCOS has been widely accepted (7, 8). Obesity could be found in 10-50% of women with PCOS who have a BMI outside the acceptable range of 19-25 kg/m^2^ (13); whereas, in the present study 70% of PCOS patients were overweight (BMI >25 kg/m^2^), higher than what was reported by Al-Nakash and Al-Tae’e, Pasquali et al. and Kiddy et al. (16), who found that 63.55%, 50% and 35% of women with PCOS were obese or overweighed, respectively. The explanation for our higher incidence of overweight can be attributed to food habits and life styles of Indian women. 

In our study, the IM+HA+PCO subgroup showed higher BMI, WC, HC compared to HA+PCO and controls. WHR is known to be a measure of abdominal obesity. The present study showed that IM+HA+PCO subgroup had less central obesity compared to the HA+PCO subgroup, similar to the findings of Chae SJ et al. Welt et al. and Belosi et al. (15, 17, 18). 

Homeostatic Model Assessment score (HOMA), is a good indicator of insulin resistance (IR). This HOMA score was significantly higher in the PCOS subgroups compared to controls, similar to the findings of Chae SJ et al. (15). The present study showed higher insulin level and HOMA scores in the HA+PCO (regular menstruation) subgroup compared to the IM+HA+PCO subgroup, which is inconsistent with that reported by Welt et al. (18), who found higher insulin levels in those with irregular menstruation. This increased IR can be explained due to higher central obesity in the HA+PCO group similarly explained by Cosar et al. (19). Dyslipidemia is one of the common metabolic abnormalities in PCOS, which is associated with IR and hyperinsulinemia, which was also noted in our study (1, 2).

In our study, increased LH, FSH and decreased LH/FSH ratio were noted in the HA+PCO group compared with the IM+HA+PCO subgroup and controls (Table 2). This difference can be explained by the presence of central obesity. Both LH and insulin stimulate androgen production in women with PCOS (20), and both have been suggested as the primary factors related to weight and anovulation that stimulate ovarian androgenesis. IM+HA+PCO (n = 112, 61%) and HA+PCO (n = 16, 69%) showed LH/FSH ratio of more than 2, which was significantly different compared to the controls, similar to the inferences by Al-Nakash and Al-Tae’e (21), but contrary to Cho et al. (16), who stated that LH/FSH ratio had limited use in the diagnosis of PCOS. 

Phenotypic study based on BMI: overweight or obesity affects approximately 60-80% of PCOS patients (22). Similarly, we had 142 (70%) obese PCOS and 62 (30%) lean PCOS based on the Asia-Pacific definition, BMI ≥ 25 and < 25 kg/m2, respectively (6, 7). The magnitude of overweight and obesity is directly related to insulin resistance in PCOS patients (23). Obese PCOS subgroup had more fasting insulin, fasting glucose, and HOMA score (IR) values compared to lean PCOS and controls. Also obese PCOS showed a significant difference in LH, FSH, LH/FSH, TG, and HDL values compared to lean PCOS and controls.

Finally, metabolic syndrome (MetS) has been over-emphasized in PCOS regarding its therapeutic approach. The IM+HA+PCO subgroup showed greater prevalence of MetS compared to HA+PCO and controls; similarly Welt et al. (18), showed highest prevalence for the IM+HA group. Amato et al. (24) compared PCOS patients according to the Rotterdam, AES and NIH criteria, and found that, regardless of the diagnostic criteria used, metabolic parameters and insulin sensitivity are more important for correct diagnosis and treatment of PCOS. 

The current study revealed significant differences among the phenotypic subgroups of polycystic ovarian syndrome, as evidenced by their anthropometric, biochemical and ultrasonographic details, especially in women with regular menstrual cycles. Evaluation of these parameters is of paramount importance in early diagnosis of PCOS and its monitoring. 
